# Synthesis, Characterization and Catalytic Activity of Ternary Oxide Catalysts Using the Microwave-Assisted Solution Combustion Method

**DOI:** 10.3390/ma13204607

**Published:** 2020-10-16

**Authors:** Kawthar Frikha, Lionel Limousy, Jamel Bouaziz, Kamel Chaari, Simona Bennici

**Affiliations:** 1 Institut de Science des Materiaux de Mulhouse UMR 7361, Université de Haute-Alsace, Centre National de la Recherche Scientifique, F−68100 Mulhouse, France; kawthar.frikha@uha.fr (K.F.); lionel.limousy@uha.fr (L.L.); 2 Institut de Science des Materiaux de Mulhouse UMR 7361, Université de Strasbourg, F-67000 Strasbourg, France; 3 Laboratoire de Chimie Industrielle, Département de Génie des Matériaux, Ecole Nationale d’Ingénieurs de Sfax, Université de Sfax, BP1173, Sfax 3038, Tunisie; jamel.bouaziz@enis.rnu.tn (J.B.); kamel.chaari@enis.rnu.tn (K.C.)

**Keywords:** Ni−Co−Al, Ni−Cu−Al, Co−Cu−Al ternary oxide catalysts, microwave-assisted solution combustion, CO oxidation

## Abstract

Ni−Co−Al, Ni−Cu−Al and Co−Cu−Al ternary oxide catalysts, with a fixed 5 wt% transition metal loading, were prepared by the microwave-assisted solution combustion method and tested in CO oxidation. The bulk and surface properties of the catalysts were investigated, using XRD, N_2_ adsorption–desorption, SEM, XPS and TEM techniques. XRD, XPS and TEM results revealed that nickel and cobalt were present as spinels on the surface and in the bulk. Differently, copper was preferentially present in “bulk-like” CuO-segregated phases. No interaction between the couples of transition metal species was detected, and the introduction of Cu-containing precursors into the Ni−Al or Co−Al combustion systems was not effective in preventing the formation of NiAl_2_O_4_ and CoAl_2_O_4_ spinels in the Ni− or Co-containing catalysts. Copper-containing catalysts were the most active, indicating that copper oxides are the effective active species for improving the CO oxidation activity.

## 1. Introduction

Transition metal oxides are potential catalysts in heterogeneous catalysis. These metal oxides possess interesting electronic surface properties, which are supposed to be beneficial for their application in catalysis [1,2,3]. These properties include the presence of cationic and anionic vacancies, the ability of transition metal cations to undergo oxidation and reduction, as well as the presence of highly mobile oxygen inside their structure [4]. Ni, Co or Cu oxide-based catalysts are among the most commonly used catalysts in heterogeneous catalysis. They have several advantages, including their availability and their low price, which make them a good alternative to expensive noble metal catalysts [5,6,7]. Ni, Co or Cu oxide-based catalysts are not widely used solely as heterogeneous catalysts due to their sensitivity to poisoning and their low thermal stability [6]. Several research studies were conducted to establish strategies for improving the stability and activity of single metal oxide catalysts. These strategies consist of dispersing the oxides on various catalyst supports, doping the oxides with metal additives or producing mixed oxide structures [8,9,10,11,12,13,14,15]. The use of mixed oxide catalysts seems to promote the catalytic activity. Indeed, transition metal elements with mixed valences are beneficial to enhance the electronic, structural, and chemical properties and to obtain high catalytic performances [4].

The solution combustion route is a well-established method to prepare mixed oxide catalysts. This method has been successfully applied to prepare Ni–, Cu– or Co−γ−Al_2_O_3_ catalyst systems [16,17,18,19,20]. These studies have assisted in providing valuable insights into the bulk and surface properties of the prepared catalysts. In our previous works [16,17], it has found that Cu oxides do not interact with γ−alumina and develop three dimensional crystallites, whose properties are similar to those of large bulk crystals. Differently, Ni or Co oxides interact with γ−alumina to form MAl_2_O_4_ “surface spinels” and MAl_2_O_4_ solid solution. This interaction derives from the incorporation of metal ions into the alumina lattice sites of octahedral or tetrahedral symmetry. Similar solid–solid interaction has also been observed for similar catalyst systems prepared by other methods, such as impregnation and co-precipitation [21,22,23,24,25,26,27]. The activity of Ni–, Cu– or Co−γ−Al_2_O_3_ catalyst systems in the total oxidation of CO has been extensively studied. In most cases, the formation of metal aluminates species was accompanied with a significant decrease in CO oxidation activity [17]. This trend was explained by the fact that the solid–solid interaction between γ−Al_2_O_3_ and the transition metal oxide affects the state of the transition metal, thus affecting the catalytic activity in CO oxidation. One of the currently used solutions to improve the catalytic performance of the Ni–, Cu– or Co−γ−Al_2_O_3_ catalyst systems is the addition of rare-earth metal oxides (CeO_2_, La_2_O_3_) or alkaline earth metal oxide (MgO, CaO) as promoters [9,10,11,14,15]. Another method has been proposed is the addition of a second active metal/metal oxide phase into the catalyst. For example, You et al. [28] prepared Ni−Co/γ−Al_2_O_3_ catalysts for CH_4_ steam reforming, and studied the effect of Co addition onto their catalytic performances. The authors detected the formation of Ni–Co alloy in the reduced catalyst, which improved the catalytic performances. In another study, Wang et al. [29] reported that Ni–Co/Al_2_O_3_ catalysts possessed a much higher catalytic activity in the steam reforming of biomass tar than those of Ni/Al_2_O_3_ or Co/Al_2_O_3_ catalysts. Lu et al. [30] studied the effects of the Cu/Co ratio on the performance of reduced Cu−Co/Al_2_O_3_ catalysts for volatile organic compound (VOC) oxidation. They found that the addition of a small amount of Cu metal (1.68 wt%) to the catalyst enhanced the dispersion of metal particles and promoted the catalytic reaction. Sagata et al. [31] studied the effect of CoO_x_ or NiO_x_ additives on the performance of Al_2_O_3_-supported Cu catalysts for the low-temperature water–gas shift (LT-WGS) reaction. It was shown that the catalytic activity of Cu/Al_2_O_3_ catalyst was improved by CoO_x_ or NiO_x_ addition, and the Cu−Co/Al_2_O_3_ catalyst showed the highest activity. In the same context, Wang et al. [32] investigated the effect of Ni addition on the Cu/γ−Al_2_O_3_ catalysts’ activity and durability for dimethyl ether steam reforming. It was found that the Ni addition improved the Cu surface dispersion, strengthened the interaction between Cu and γ−Al_2_O_3_, and enhanced the copper resistance to deactivation.

The present work is a part of a broad study on the improving of the physicochemical properties of combustion-synthesized mixed oxides catalysts by the addition of a second active phase into the catalyst. The modifications were particularly oriented to inhibit the formation of NiAl_2_O_4_ and CoAl_2_O_4_ spinels in the Ni− or Co-containing catalysts. It seems that the second metal phase added to Ni or Co and γ−alumina reduces the strong Ni− or Co−γ−alumina interaction by inhibiting the incorporation of Ni or Co ions into the alumina lattice. The number of Ni or Co active sites are then increased. Moreover, the addition of Cu-based phases will generate new active sites in the Ni− or Co-containing catalysts. In this paper, X-ray diffraction (XRD), N2 adsorption–desorption, transmission electron microscope (TEM) and X-ray photoelectron spectroscopy (XPS) techniques have been used to determine the effect of the presence of a second active transition metal on the chemical state of the active species and their chemical interactions. Characterization results were correlated with the CO oxidation activity. To the best of our knowledge, the use of microwave-assisted solution combustion technology for the synthesis of Ni–Co–Al, Ni–Cu-Al and Co–Cu–Al ternary oxide catalysts has never been conducted before. In addition, investigation of CO oxidation over these catalytic materials has been rarely reported.

## 2. Materials and Methods

### 2.1. Catalyst Preparation

Aluminum nitrate nonahydrate (Al(NO_3_)_3_·9H_2_O, ≥98% Fluka, Steinheim am Albuch, Baden-Württemberg, Germany), nickel nitrate hexahydrate (Ni(NO_3_)_2_·6H_2_O, ≥99% Merck, Darmstadt, Germany), cobalt nitrate hexahydrate (Co(NO_3_)_2_·6H_2_O, ≥99% Merck) and copper nitrate trihydrate (Cu(NO_3_)_2_·3H_2_O, ≥99.5% Merck) were used as the metal precursors, as well as the oxidizing agents. Urea (CO(NH_2_)_2_, >99% Fluka) was used as the reducing agent (fuel). All reagents were used as received without any further purification. The initial proportions of all reagents were calculated based on the thermo-chemical concepts of propellant chemistry [33].

The synthesis of the three catalysts was carried out in the same manner as outlined in previous works [16,17]. At first, metal nitrate hydrates were dissolved in 10 mL of demineralized water. The quantities of the Ni, Co and Cu precursors were adjusted to give a transition metal loading of 5 wt%. Then, urea was added. The obtained solution was kept under stirring with a stirring speed of 450 rpm. The mixing was performed at 60 °C and lasted for 1 h in order to remove the excess water. Afterward, the obtained mixture was heated in a laboratory microwave oven operating at 700 W, 2.45 GHz, until reaching the initiation of the combustion reaction. After synthesis, the obtained catalyst powders were calcined in a muffle furnace at 500 °C in air atmosphere for 12h with a heating rate of 5 °C min^−1^.

The synthesis of the overall investigated samples was conducted under the optimal conditions (RV/OV = 1), considering that the best physicochemical properties are achieved when the redox system is stoichiometrically balanced [16]. The combustion-synthesized catalysts were labelled as 5M5MAl (where 5 = transition metal loading (wt%); M = Ni, Cu or Co).

### 2.2. Catalyst Characterization

Powder X-ray diffraction (XRD) patterns were recorded at room temperature on an X’Pert Pro MPD diffractometer (PANalytical, Eindhoven, The Netherlands) operating with a Cu Kα radiation source, λ = 0.15406 nm at 40 mA and 45 kV. Data were recorded in the 2 theta (2θ) range of 10−90°, in step size of 0.017°, and scan step time of 220 s.

Catalyst loadings (wt%) were determined by wavelength dispersive X-ray fluorescence (WDXRF) in a Zetium (4 kW) spectrometer (PANalytical, Eindhoven, The Netherlands).

Nitrogen adsorption–desorption isotherms were acquired at liquid nitrogen temperature (−196 °C) using an ASAP2040 apparatus (Micromeritics, Norcross, GA, USA). Prior to measurement, the powders were degassed under vacuum at 200 °C for 10 h. The specific surface areas were determined by applying the Brunauer–Emmett–Teller (BET) method. The pore volumes and the average pore diameters were determined using the standard Barrett–Joyner–Halenda (BJH) method.

Scanning electron microscopy (SEM) micrographs were acquired at 7 kV accelerating voltage, using an XL30 microscope (PHILIPS, Tokyo, Japan). Prior to the observations, the powders were spread on a carbon tape substrate.

X-ray photoelectron spectroscopy (XPS) analyses were carried out with a SES 2002 spectrometer (VG Scienta, Uppsala, Sweden), using a monochromatic Al Kα X-ray source (Al Kα = 1486.6 eV). The spectra were obtained using an analysis area of around 24 mm^2^, 100 eV pass energy and a 10^−9^ Pa pressure in the analysis chamber. The C 1s peak at 284.7 eV of contaminated carbon was used as reference to calibrate the binding energies (BEs). Gaussian–Lorentzian functions were used to fit XPS peaks with the XPS-CASA software (Casa Software Ltd., Version 2.3.18, Teignmouth, UK).

Transmission electron microscopy (TEM) measurements were performed on an ARM-200F microscope (Jeol, Tokyo, Japan), operating at 200 kV, with a probe size of about 0.08 nm. Prior to TEM measurement, the powders were dispersed in chloroform, ultra-sonicated and deposited on carbon-coated Au grid.

### 2.3. Catalytic Activity Measurements

Catalytic activity measurements were performed in a vertical fixed bed reactor, externally heated by an electric furnace. Catalytic tests were conducted under both transient and stationary conditions at atmospheric pressure. First, 40−60 mesh particle size fractions of the sieved catalysts were introduced into the reactor and heated from ambient temperature to the desired reaction temperature, at a heating rate of 5 °C min^−1^, under a gas stream of 0.05 vol% CO and 1 vol% O_2_ in N_2_. During the experiments, the inlet gas flow rate was adjusted at 50 NLh^−1^, and the space velocity (GHSV) was maintained at 3500 mLgcat^−1^h^−1^. The outlet CO concentration was analyzed on-line by an infrared NGA 2000 gas analyzer (Emerson Process Management, Hasselroth, Germany) and recorded each minute using the MLT Analyzer software (Version 3.6.X, Emerson Process Management, Hasselroth, Germany). The CO conversion yield (%) was calculated by Equation (1):

CO conversion % = (([CO]_in_ − [CO]_out_)/[CO]_in_) × 100, (1)

where [CO]_in_ is the CO inlet gas concentration and [CO]_out_ is the CO outlet gas concentration.

## 3. Results

### 3.1. Physico-Chemical Characterization

#### 3.1.1. XRD

**Figure 1:**
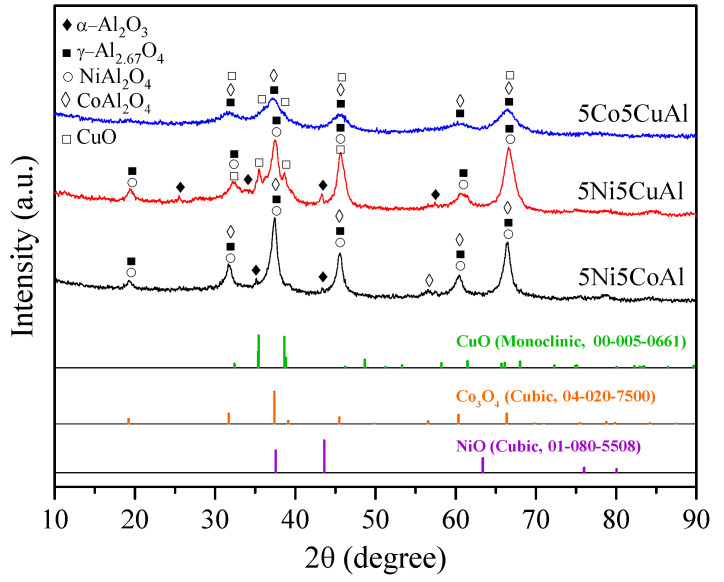
XRD patterns of the 5M5MAl catalyst samples.

The XRD diffractograms of the 5M5MAl catalyst samples are presented in Figure 1. The XRD patterns of the 5Ni5CoAl catalyst showed broad and overlapped diffraction peaks at 2θ = 31.7°, 37.5°, 45.7°, 60.3° and 66.4°, which were assigned to γ−Al_2.67_O_4_ (JCPDS 04−005−4662), CoAl_2_O_4_ (JCPDS 00−003−0896), and NiAl_2_O_4_ (JCPDS 01−078−6951). Diffraction peaks related to α−Al_2_O_3_ (JCPDS 00−042−1468) at 2θ = 35.1° and 43.4° were also detected. There is, however, no diffraction peak related to NiO or Co_x_O_y_ phases. The XRD patterns of the 5Ni5CuAl catalyst showed, major diffraction peaks of γ−Al_2.67_O_4_ (JCPDS 04−005−4662) and NiAl_2_O_4_ (JCPDS 01−078−6951). The depicted peaks at 2θ = 25.5°, 35.5°, 43.4° and 57.2° were assigned to α−Al_2_O_3_ (JCPDS 00−042−1468). Moreover, well defined peaks of CuO (JCPDS 00−005−0661) were detected. Similarly, on the 5Ni5CoAl catalyst, no peaks related to the existence of NiO were detected. For the 5Co5CuAl catalyst, the peaks at 2θ = 31.5°, 37.1°,45.6°, 60.3° and 66.5° were assigned to γ−Al_2.67_O_4_ (JCPDS 04−005−4662) and CoAl_2_O_4_ (JCPDS 00−003−0896) phases. It was difficult to distinguish between γ−Al_2.67_O_4_ and CoAl_2_O_4_ phases from XRD measurements, because their diffraction patterns are superposed. Additionally, weak diffraction peaks related to CuO (JCPDS 00−005−0661) were observed. It can be expected that the dispersion of CuO in the 5Co5CuAl sample is slightly higher than that for the 5Ni5CuAl sample. No crystalline phases corresponding to Co_x_O_y_ could be observed. The absence of any diffraction peaks of NiO or Co_x_O_y_ phases, in Ni− or Co-containing catalysts, indicates the diffusion of cobalt and nickel into the γ−Al_2.67_O_4_ lattice, producing NiAl_2_O_4_ and CoAl_2_O_4_ phases [16,17]. Meanwhile, no detectable peaks of alloy systems were observed on the three studied samples, suggesting that there is no interaction between the couples of transition metal species. These initial observations were further explored by performing TEM and XPS characterizations.

XRD results reveal that a defect spinel γ-alumina (γ-Al_2.67_O_4_) and metal aluminates MAl_2_O_4_ (where M = Ni or Co) were the mainly phases formed in the 5Ni5CoAl catalyst. The 5Ni5CuAl catalyst consists of well-crystallized CuO and NiAl_2_O_4_ phases, together with the γ−Al_2.67_O_4_ phase, while the 5Co5CuAl catalyst consists of poorly crystallized CuO, CoAl_2_O_4_ and γ−Al_2.67_O_4_ phases. XRD results of the 5Ni5CoAl catalyst were quite different from those reported in previous studies. Sajjadi et al. [34] prepared Ni−Co/Al_2_O_3_ catalyst by the sol–gel method. The authors revealed the formation of NiAl_2_O_4_, Co_3_O_4_ and NiO phases. The XRD results of Cinar et al. [35] showed Ni−Cu alloy formation in Al_2_O_3_-supported Ni−Co-based catalyst using the polyol process. Sajjadi et al. [36] registered the formation of γ−Al_2_O_3_, Co_3_O_4_ and NiO phases in a Co-doped Ni/Al_2_O_3_ catalyst using the sol–gel method. You et al. [28] prepared Ni−Co/γ−Al_2_O_3_ catalysts by the impregnation method. They detected the formation of NiO, Co_3_O_4_, NiAl_2_O_4_ and CoAl_2_O_4_ phases. The XRD results of the 5Ni5CuAl catalyst differ from those obtained by other preparation methods. Khzouz et al. [37] detected the presence of Ni_x_Cu_1_–_x_O, γ−Al_2_O_3_ and traces of θ−Al_2_O_3_ in 5wt%Ni−5wt%Cu/Al_2_O_3_ catalysts, prepared by the impregnation method. Zhang et al. [38] found that, for the 30wt%Ni−5wt%Cu/γ−Al_2_O_3_ catalyst prepared by precipitation impregnation, only diffraction peaks of NiO and γ−Al_2_O_3_ were present. The results of Sajjadi et al. [36] showed the formation of NiAl_2_O_4_, CuO and NiO phases in Ni−Cu/Al_2_O_3_ catalyst using the sol–gel method. Rahemi et al. [39] detected the presence of NiAl_2_O_4_, γ−Al_2_O_3_ and NiO phases in 10wt%Ni−3wt%Cu/Al_2_O_3_ nanocatalysts synthesized via the impregnation method. In comparison to Ni−Co−Al and Ni−Cu−Al-based mixed oxide catalysts, only few references can be found dealing with the study of Co−Cu−Al-based mixed oxide catalyst; for example, Lu et al. [30] used the polyol process to generate 1 wt%Cu−1 wt%Co/Al_2_O_3_ catalyst. The authors detected the presence of γ−Al_2_O_3_, Cu and CuO phases.

#### 3.1.2. N_2_ Adsorption–Desorption Isotherms

**Figure 2:**
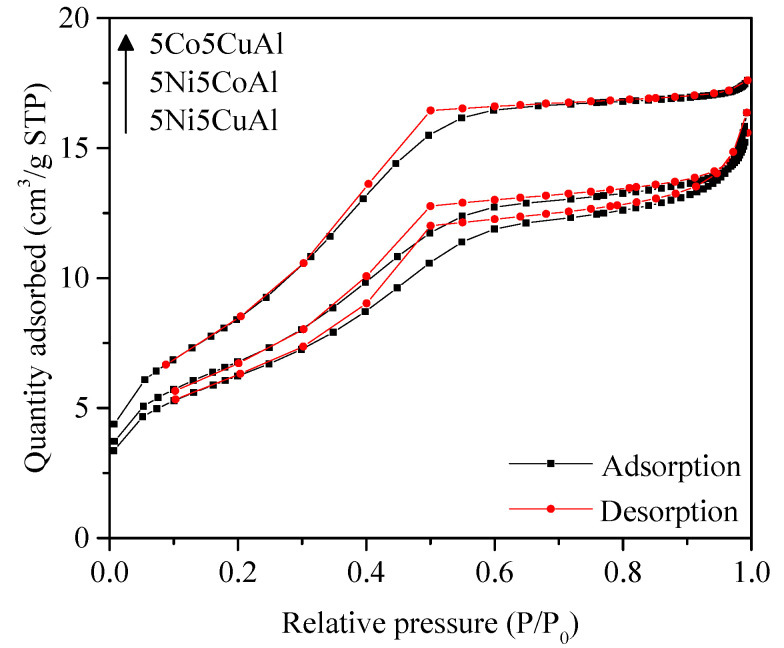
N_2_ adsorption–desorption isotherms of the 5M5MAl catalyst samples.

The N_2_ adsorption–desorption isotherms of the 5M5MAl catalyst samples are shown in Figure 2. The isotherms of 5M5MAl samples are of type IV, characteristic of mesoporous solids, with H4-type hysteresis loops. The textural properties are summarized in Table 1. All the samples were found to have relatively low surface areas, ranging from 23 to 31 m^2^g^−1^. The low surface area of the ternary oxide catalysts can be directly correlated to the alumina phase transformation, which is considered as an important factor for determining the textural properties of the catalyst. The major phases detected into the three studied samples were metal aluminates (MAl_2_O_4_). According to the literature [40], the surface areas of the combustion-synthesized metal aluminate (MAl_2_O_4_) structures are generally low and thus result in a low surface area of the synthesized catalysts. These ternary oxide catalysts were also found to have lower surface areas than the binary oxide catalysts, as reported in our previous work [16,17].

#### 3.1.3. SEM

**Figure 3:**
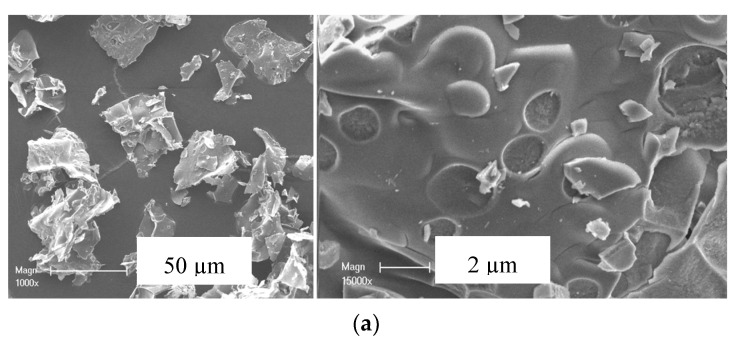

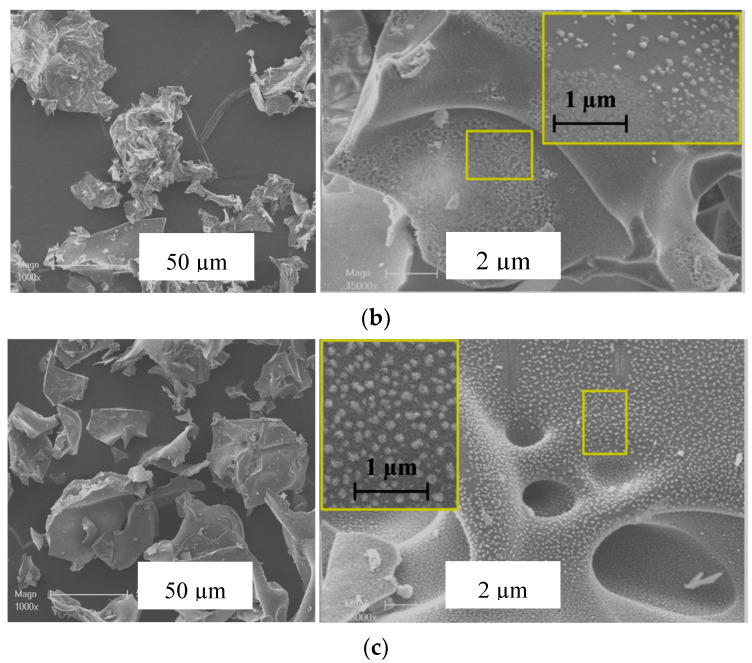
SEM images of the (**a**) 5Ni5CoAl, (**b**) 5Ni5CuAl and (**c**) 5Co5CuAl catalyst samples.

The SEM micrographs of the 5M5MAl catalyst samples are illustrated in Figure 3. It can be seen that the synthesized catalysts have “flaky-like” morphology with an average size of agglomerates higher than 50 µm. Variation in surface features was found between the different three samples. The 5Ni5CoAl sample presented a soft surface, while quasi-spherical nanoparticles, in the range of 20−100 nm, were dispersed on the surface of the 5Ni5CuAl and 5Co5CuAl samples. These nanoparticles could be assigned to the copper oxide nanoparticles (confirmed by SEM-EDX analysis—not shown). The area enclosed by the rectangle reveals that the 5Co5CuAl sample (Figure 3c) exhibited more uniform particle distribution with regular shapes and without clear agglomerated particles.

#### 3.1.4. TEM and EDX Analysis

**Figure 4:**
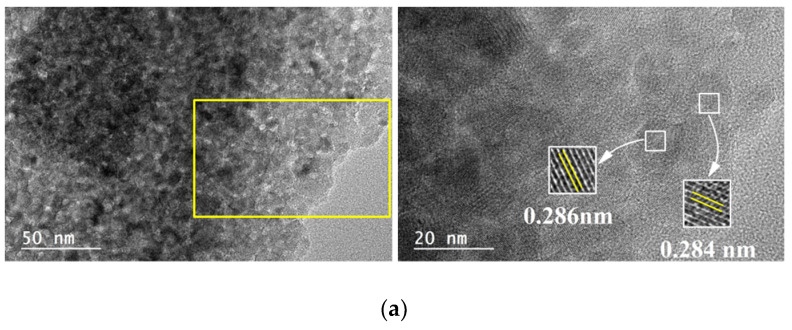

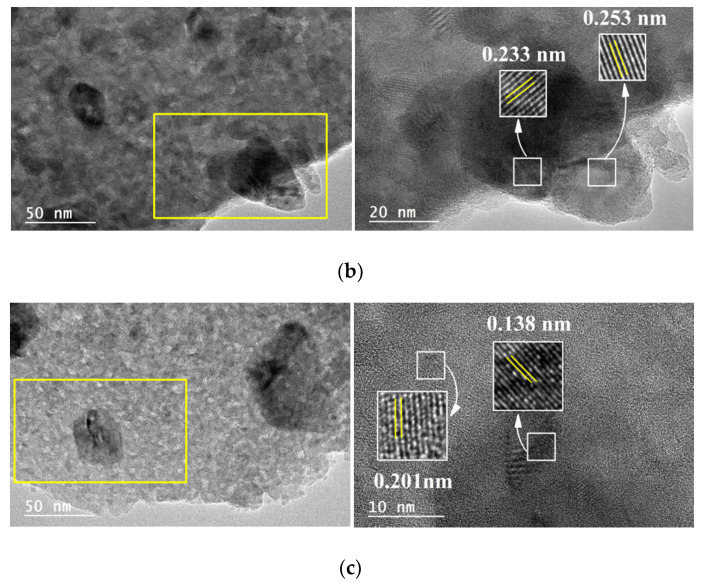
TEM images of the (**a**) 5Ni5CoAl, (**b**) 5Ni5CuAl and (**c**) 5Co5CuAl catalyst samples.

The TEM micrographs of the 5M5MAl catalyst samples are shown in Figure 4. Ni, Co and Cu metal oxide nanoparticles can be easily identified on the surface of the catalysts due to the color contrast between the metal oxide and alumina. As it can be seen from Figure 4a, Ni and Co metal oxide nanoparticles were uniformly dispersed on the surface of the 5Ni5CoAl catalyst, with an average particle size in the 5–20 nm range. Figure 4a also shows many crystal lattice planes of the 5Ni5CoAl catalyst with d-spacing of 0.284 and 0.286 nm, corresponding to the cubic NiAl_2_O_4_ (220) plane and to the cubic CoAl_2_O_4_ (220) plane, respectively. Therefore, the homogeneous dispersion of the Ni and Co metal oxide nanoparticles in 5Ni5CoAl catalyst was attributed to the formation of well-dispersed (monolayer structures) NiAl_2_O_4_ and CoAl_2_O_4_ “surface spinels”. According to Figure 4b,c, three dimensional CuO nanoparticles, ranging from 20 to 100 nm in size, were identified in both 5Ni5CuAl and 5Co5CuAl catalysts. Slightly larger CuO particles and an apparent agglomeration are observed in the 5Ni5CuAl catalyst. Longer CuO nanoparticles were observed on these two samples and attributed to sintering and agglomeration phenomena. d-spacings of 0.233 and 0.253 nm, corresponding to the CuO (111) and (002) planes, respectively, were measured for the 5Ni5CuAl catalyst, while the 5Co5CuAl catalyst presented lattice planes with d-spacing of 0.138 and 0.201 nm for the CuO (220) plane and the CoAl_2_O_4_ (400) plane, respectively.

**Figure 5:**
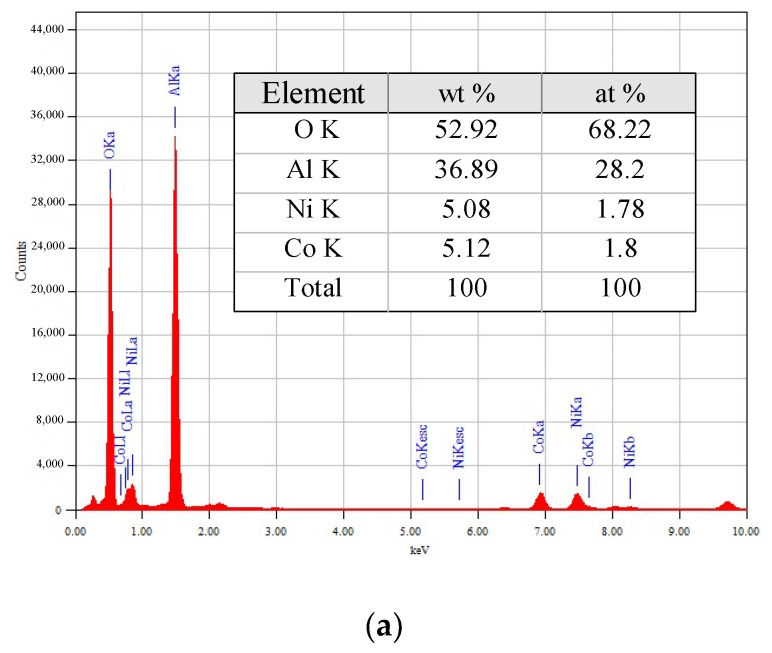

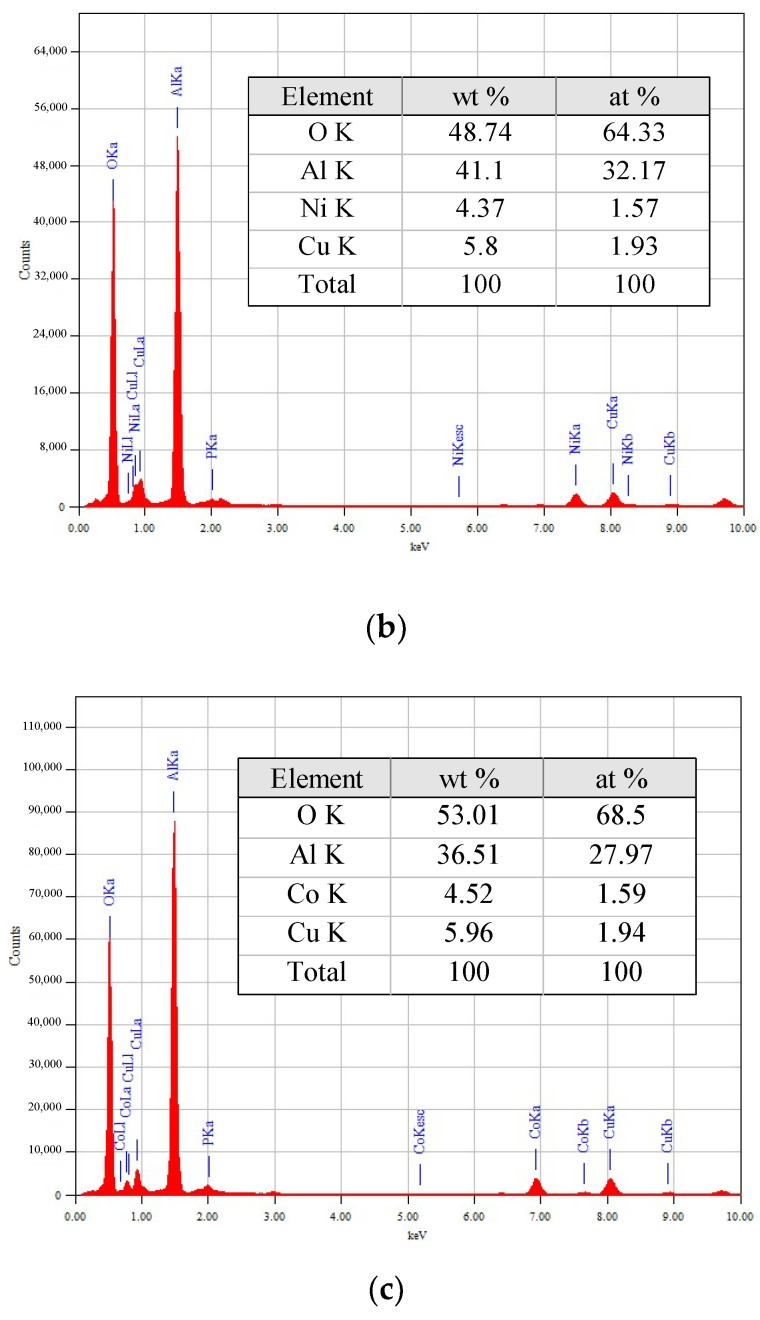
TEM-EDX elemental analyses of the (**a**) 5Ni5CoAl (**b**) 5Ni5CuAl and (**c**) 5Co5CuAl catalyst samples.

EDX spectra and elemental compositions of the 5M5MAl samples are reported in Figure 5. The metal loadings (wt%) obtained from EDX analyses were similar to the nominal (5 wt%). Obviously, the quantitative amounts of the copper in the 5Co5CuAl and 5Ni5CuAl catalyst samples are slightly higher than the actual metal loadings, suggesting that copper species is mostly present on the catalyst surface.

**Figure 6:**
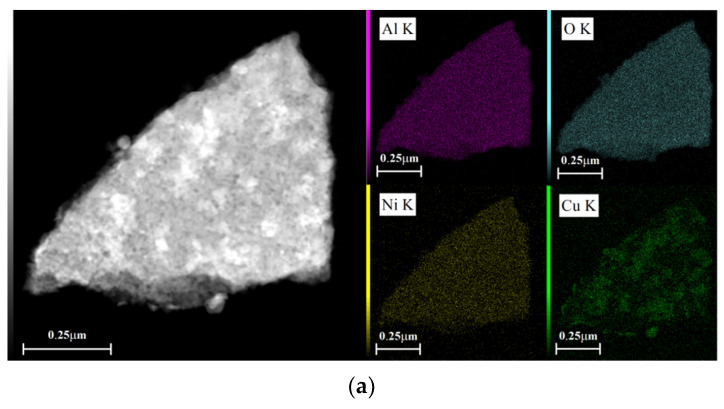

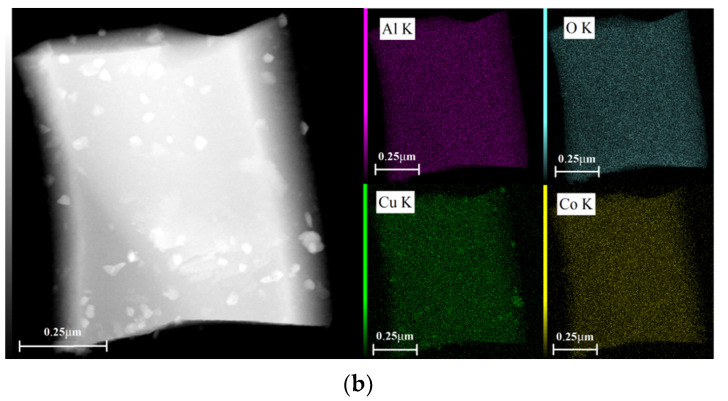
TEM-EDX dot-mapping analyses of the (**a**) 5Ni5CuAl and (**b**) 5Co5CuAl catalyst samples.

Figure 6 shows EDX dot-mapping analyses of 5Ni5CoAl and 5Ni5CuAl samples. Figure 6a,b show that Ni and Co elements are homogeneously dispersed on the surface of 5Ni5CuAl and 5Co5CuAl samples, respectively. Differences were enlightened on the Cu dispersion over the two samples: a highest local Cu concentration was observed on the surface of 5Co5CuAl sample. These results are consistent with the observations of TEM micrographs that show large particles of CuO and an apparent agglomeration of CuO nanoparticles in the 5Co5CuAl sample.

**Figure 7:**
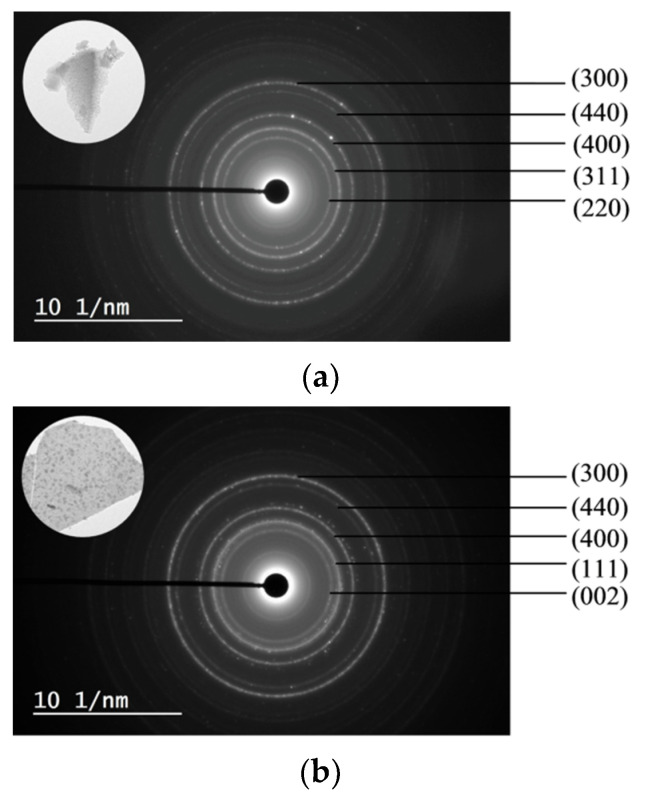

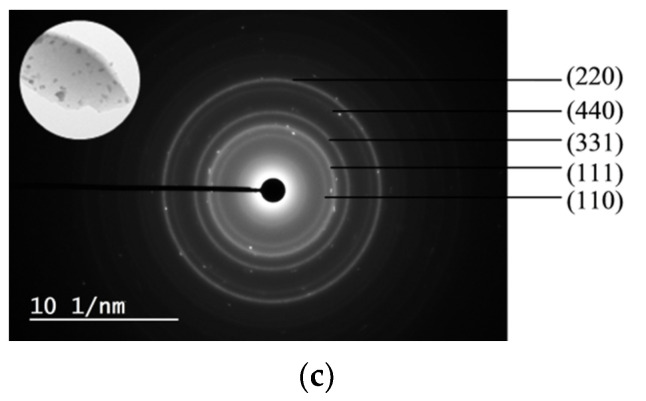
SAED patterns of the (**a**) 5Ni5CoAl (**b**) 5Ni5CuAl and (**c**) 5Co5CuAl catalyst samples.

SAED patterns and their corresponding TEM images (inset) are shown in Figure 7. The SAED patterns of the 5Ni5CoAl sample consist of continuous concentric rings that could be assigned to the α−Al_2_O_3_ (300) plane, the CoAl_2_O_4_ (440) plane, and to the NiAl_2_O_4_ (400), (311) and (220) planes. For the 5Ni5CuAl sample, the diffraction spot forms concentric rings corresponding to the α−Al_2_O_3_ (300) plane, NiAl_2_O_4_ (440) and (400) planes, and to the CuO (111) and (002) planes. For the 5Co5CuAl sample, five diffraction rings are observed, which could be indexed as the CuO (220), (111) and (110) planes, as well as the CoAl_2_O_4_ (440) and (331) planes. TEM results suggest the presence of NiAl_2_O_4_ or CoAl_2_O_4_ “surface spinels” in the Ni− or Co-containing catalysts, while the formation of “bulk-like” CuO nanoparticles in the Cu-containing catalysts was confirmed. These observations were associated to the well-known interaction between alumina and transition metal oxides. Indeed, incorporated nickel or cobalt oxides develop two-dimensional (2D) nanostructures, forming well-dispersed CoAl_2_O_4_ and NiAl_2_O_4_ “surface spinels”. On the other hand, copper oxides that do not interact with alumina develop three dimensional (3D) crystallites. Neither nickel and cobalt oxides nor alloy systems were observed in all the samples, which is in accordance with the XRD results.

#### 3.1.5. XPS

**Figure 8:**
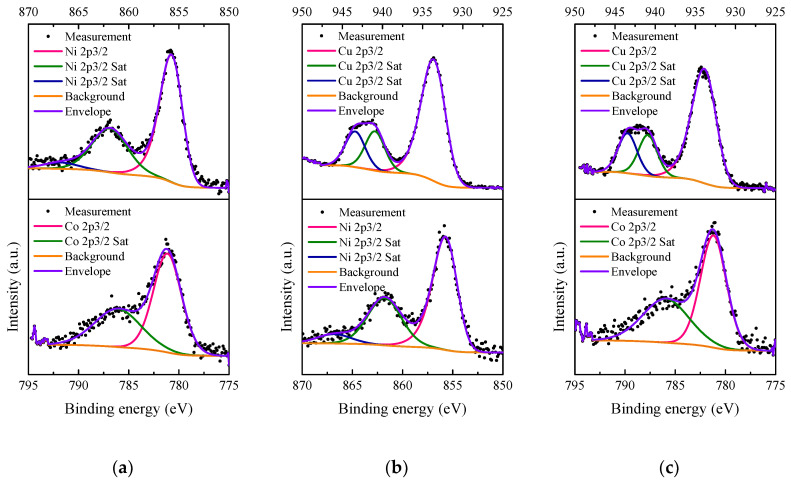
XP spectra of the (**a**) 5Ni5CoAl, (**b**) 5Ni5CuAl and (**c**) 5Co5CuAl catalyst samples.

The Ni 2p_3/2_, Co 2p_3/2_ and Cu 2p_3/2_ XP spectra of 5M5MAl catalyst samples are presented in Figure 8. As shown in Figure 8a, the Ni 2p_3/2_ signal is composed of a main band centered at 855.34 eV, accompanied by two satellites at BEs of 861.77 and 866.8 eV, and assigned to Ni^2+^ in NiAl_2_O_4_ [41,42]. The presence of Co^2+^ in CoAl_2_O_4_ was also confirmed by the Co 2p_3/2_ peak at 781.14 eV, accompanied by the satellite peak at around 786.09 eV [43,44]. Compared with our previous XPS studies on the binary oxide catalysts [16,17], the combination of Ni− and Co-based catalysts in the 5Ni5CoAl ternary oxide catalyst does not seem to affect the positions of both Ni 2p_3/2_ and Co 2p_3/2_peaks. This further confirms the existence of NiAl_2_O_4_ and CoAl_2_O_4_ compounds on the 5Ni5CoAl catalyst surface. For the 5Ni5CuAl catalyst sample (Figure 8b), the Ni 2p_3/2_ signal shows a peak at 855.35 eV with two satellite peaks at BEs of 861.78 and 866.8 eV. Ni peak positions are exactly the same for 5Ni5CoAl and 5Ni5CuAl, showing that the Ni species were in the same oxidation state in both samples. The Cu 2p_2/3_ signal showed a peak at 933.17 eV and two satellite peaks at BEs of 940.93 and 943.39 eV, corresponding to Cu^2+^ in CuO [45,46]. The Co 2p_3/2_ spectra of 5Co5CuAl catalyst (Figure 8c) showed a peak at BE of 780.57 eV, accompanied by a satellite peak at 786.21 eV, corresponding to the Co^2+^ in CoAl_2_O_4_. The Cu 2p_2/3_ spectra presented a peak at 933.43 eV, accompanied by two satellite peaks at BEs of 940.9 and 943.44 eV, and ascribed to Cu^2+^ in CuO.

XPS results suggest that Ni and Co oxides were present as NiAl_2_O_4_ and CoAl_2_O_4_ “surface spinels” on the surface of the Ni− or Co-containing catalysts, while copper oxides exist in the CuO form on the surface of the Cu-containing catalysts, without interacting with alumina, which is in accordance with the TEM results.

Surface M/Al (M = Ni, Co, Cu) atomic ratios determined by XPS of the various catalysts are summarized in Table 2. According to Table 2, the surface Ni/Al and Co/Al atomic ratios for the 5Ni5CoAl catalyst are found to be 0.035 and 0.033, respectively. The Cu/Al ratios of 5Ni5CuAl and 5Co5CuAl samples are consistently higher than the values obtained for Ni/Al and Co/Al ratios, which indicates the existence of higher amounts of Cu^2+^ on the surface compared to Ni^2+^ and Co^2+^. The highest surface Cu/Al atomic ratio was obtained with the 5Ni5CuAl catalyst sample, indicating a higher Cu concentration on the upper surface layers of the 5Ni5CuAl catalyst, which may favor the CO oxidation activity.

#### 3.1.6. Catalytic Tests

**Figure 9:**
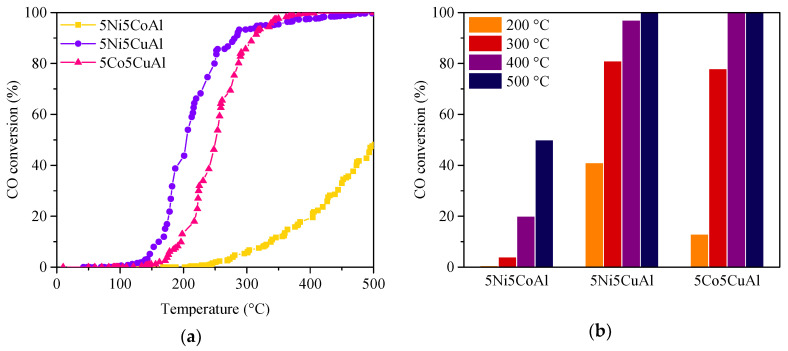
(**a**) CO conversion under transient and (**b**) steady-state conditions over the 5M5MAl catalyst samples.

The catalytic performances of the 5M5MAl catalyst samples in the model reaction of CO oxidation to CO_2_ are presented in Figure 9. To compare the activity of the various samples, the light-off temperatures of the 5M5MAl catalysts are listed in Table 3. The oxidation of CO over the 5Ni5CuAl and 5Co5CuAl catalysts began at 125 and 140 °C, respectively. Differently, 5Ni5CoAl started converting CO only at 243 °C. The catalytic activity of the 5Ni5CuAl and 5Co5CuAl samples rapidly increased with the temperature, reaching 50% conversion at 208 and 268 °C, respectively. Moreover, 100% conversion was reached at 400 °C. On the other hand, the activity of 5Ni5CoAl sample increased more progressively, giving 50% conversion at approximately 500 °C. This catalyst did not reach the total conversion of CO in the investigated temperature range (Figure 9a). Among the three investigated catalysts, 5Ni5CoAl showed to be the less active. The performance of 5Ni5CuAl catalyst was slightly greater than that of 5Co5CuAl in the temperature range from 100 °C to 300 °C, while similar conversion profiles were obtained at temperature higher than 300 °C for these two catalysts.

Figure 9b compares the CO conversion for the three catalysts under steady state conditions. CO oxidation activity for the 5Ni5CoAl catalyst is considerably weak at low and high temperature values, giving a CO conversion of approximately 20% at 400 °C and a maximum value of 50% at 500 °C. The poor CO oxidation activity of the 5Ni5CoAl sample could be attributed to the low concentration of nickel and cobalt in the top surface layers of the catalyst as well as to their presence as NiAl_2_O_4_ and CoAl_2_O_4_ phases. The 5Ni5CuAl catalyst showed a higher CO oxidation activity than the 5Co5CuAl catalyst at 200 °C: 41% and 13% of CO conversions were obtained for 5Ni5CuAl and 5Co5CuAl at 200 °C, respectively. The CO conversion values for both 5Ni5CuAl and 5Co5CuAl catalysts were almost the same at 300, 400 and 500 °C. The CO oxidation activity of 5M5CuAl catalysts could be correlated to the degree of crystallization of CuO crystallites and to the surface concentration of copper oxide. It is seen that the 5Ni5CuAl catalyst consists of well-crystallized CuO phases, while the 5Co5CuAl catalyst consists of very poorly crystallized CuO. Moreover, the concentration of copper on the upper surface layers of the 5Ni5CuAl catalyst is higher than that on the 5Co5CuAl catalyst. This can explain the best catalytic performances of the 5Ni5CuAl catalyst at low temperature for CO oxidation.

## 4. Conclusions

The combustion method is a simple and fast synthesis route for the preparation of Ni−Co−Al, Ni−Cu−Al and Co−Cu−Al ternary oxide catalysts. The bulk and surface characterization results showed that nickel and cobalt species form “surface spinels” and “bulk” metal aluminate (MAl_2_O_4_) phases in the Ni− or Co-containing catalysts, while copper species preferentially form “bulk-like” CuO segregated phases in the Cu-containing catalysts. The comparison study between the catalytic activities of the different ternary oxide catalysts showed that 5Ni5CuAl and 5Co5CuAl catalysts were more active than 5Ni5CoAl catalysts, and the 5Ni5CuAl catalyst was the most effective. Highly dispersed CuO species on the catalysts’ upper surface layers, and well-crystallized CuO phases were responsible for the high CO oxidation activity. For the 5Ni5CoAl catalyst, the low activity was correlated to the formation of low active CoAl_2_O_4_ and NiAl_2_O_4_ spinels, resulting from the Ni– or Co−γ−alumina solid–solid interaction

Although the introduction of Cu-containing precursors did not succeed in inhibiting the formation of NiAl_2_O_4_ and CoAl_2_O_4_ spinels, this attempt gave more insights on the behavior of the different cations during the microwave-assisted combustion preparation. In a more complex mixture than that used in our previous work [16,17], copper cations showed a low diffusivity in the reaction media, leading to the formation of small and well-dispersed CuO nanoparticles, which are catalytically active species. On the other hand, Co and Ni cations showed a high diffusivity in the reaction media to form less active spinel phases.

## Figures and Tables

**Table 1:** X-ray fluorescence (XRF) elemental analysis and textural properties of the 5M5MAl catalyst samples.

Sample Identity	Metal Loading (wt%) ^1^			BET Surface Area (m^2^g^−1^)	Pore Diameter (nm) ^2^	Pore Volume (cm^3^g^−1^) ^3^
	Ni	Co	Cu			
5Ni5CoAl	5.60	5.66	−	25	3.60	0.03
5Ni5CuAl	5.22	−	5.88	23	4.00	0.03
5Co5CuAl	−	6.65	6.06	31	3.10	0.03

Note: ^1^ XRF elemental analysis. ^2^ BJH desorption average pore diameter. ^3^ BJH desorption pore volume.

**Table 2:** Surface M/Al atomic ratios determined by XPS for the 5M5MAl catalyst samples.

Sample Identity	Surface Atomic Ratio		
	Ni/Al	Co/Al	Cu/Al
5Ni5CoAl	0.035	0.033	−
5Ni5CuAl	0.032	−	0.136
5Co5CuAl	−	0.038	0.076

**Table 3:** Light-off, T_50_ and T_100_ temperatures of the 5M5MAl catalyst samples.

Sample Identity	T_1_	T_50_	T_100_
5Ni5CoAl	243	500	−
5Ni5CuAl	125	208	475
5Co5CuAl	140	268	369
